# Supplementary material for: Usual gait speed is inversely associated with depression in middle-aged and older adults: A cross-sectional study in Korea
Source: PLoS One. 2026 Feb 9;21(2):e0338458. doi: 10.1371/journal.pone.0338458 (PMC12885282; doi:10.1371/journal.pone.0338458)
Supplement: S1 Table — (DOCX) [file pone.0338458.s001.docx]

**S1 Table.** Participants’ characteristics based on sex-specific tertiles of UGS.

| **Variables** | **Low UGS**  (n=804) | **Mid UGS**  (n=811) | **High UGS**  (n=804) | ***p*-value** |
| --- | --- | --- | --- | --- |
| **Age** (years) | 72.47 ± 7.36 ^b,c^ | 66.27 ± 7.42 ^a,c^ | 62.45 ± 6.37 ^a,b^ | <0.0001 |
| **Male sex**, n (%) | 354 (44.03) | 356 (43.90) | 355 (44.15) | 0.99 |
| **Marital status**, n (%) |  |  |  | <0.0001 |
| Divorced/widowed/single | 247 (30.72) | 123 (15.17) | 98 (12.19) |  |
| Married/partnered | 557 (69.28) | 688 (84.83) | 706 (87.81) |  |
| **Education level**, n (%) |  |  |  | <0.0001 |
| ≤Elementary school | 524 (65.17) | 349 (43.03) | 219 (27.24) |  |
| Middle/high school | 266 (33.09) | 431 (53.15) | 544 (67.66) |  |
| ≥College | 14 (1.74) | 31 (3.82) | 41 (5.10) |  |
| **Household income**, n (%) |  |  |  | <0.0001 |
| <1 (million KRW/month) | 559 (69.53) | 349 (43.03) | 223 (27.74) |  |
| 1–<2 | 133 (16.54) | 201 (24.79) | 223 (27.74) |  |
| 2–<3 | 58 (7.21) | 110 (13.56) | 149 (18.53) |  |
| 3–<4 | 31 (3.86) | 85 (10.48) | 92 (11.44) |  |
| ≥4 | 23 (2.86) | 66 (8.14) | 117 (14.55) |  |
| **Drinking habit**, n (%) |  |  |  | 0.06 |
| Never drinker | 457 (56.84) | 426 (52.53) | 427 (53.11) |  |
| Former drinker | 81 (10.08) | 64 (7.89) | 68 (8.46) |  |
| Current drinker | 266 (33.08) | 321 (39.58) | 309 (38.43) |  |
| **Smoking habit**, n (%) |  |  |  | 0.57 |
| Never smoker | 531 (66.05) | 529 (65.23) | 525 (65.30) |  |
| Former smoker | 188 (23.38) | 177 (21.82) | 190 (23.63) |  |
| Current smoker | 85 (10.57) | 105 (12.95) | 89 (11.07) |  |
| **Regular exercise**, n (%) |  |  |  | <0.0001 |
| <150 min/week | 714 (88.81) | 661 (81.50) | 632 (78.61) |  |
| ≥150 min/week | 90 (11.19) | 150 (18.50) | 172 (21.39) |  |
| **UGS** (m/s) | 0.68 ± 0.13 ^b,c^ | 0.90 ± 0.07 ^a,c^ | 1.12 ± 0.13 ^a,b^ | <0.0001 |
| **SGDS-K score** | 4.15 ± 4.21 ^b,c^ | 2.68 ± 3.35 ^a,c^ | 2.15 ± 2.93 ^a,b^ | <0.0001 |
| **BMI** (kg/m^2^) | 24.44 ± 3.70 | 24.57 ± 3.22 | 24.49 ± 3.03 | 0.73 |
| **Waist circumference** (cm) | 90.63 ± 9.74 ^c^ | 90.00 ± 8.25 | 89.15 ± 7.80 ^a^ | <0.01 |
| **SBP** (mmHg) | 128.10 ± 16.73 ^b,c^ | 125.61 ± 16.61 ^a,c^ | 121.93 ± 15.29 ^a,b^ | <0.0001 |
| **DBP** (mmHg) | 76.89 ± 9.80 ^b^ | 78.25 ± 9.76 ^a^ | 77.76 ± 9.37 | <0.05 |
| **T-Chol** (mg/dL) | 175.29 ± 33.18 ^b,c^ | 181.46 ± 34.35 ^a^ | 183.84 ± 33.80 ^a^ | <0.0001 |
| **HDL-C** (mg/dL) | 45.38 ± 12.14 | 45.92 ± 12.36 | 45.86 ± 11.94 | 0.62 |
| **TG** (mg/dL) | 127.66 ± 69.38 ^c^ | 133.94 ± 87.60 | 138.98 ± 102.60 ^a^ | <0.05 |
| **FBG** (mg/dL) | 102.95 ± 28.80 ^b,c^ | 100.13 ± 22.50 ^a,c^ | 101.58 ± 25.99 ^a,b^ | 0.09 |
| **Hypertension**, n (%) | 487 (60.57) ^b,c^ | 442 (54.50) ^a,c^ | 369 (45.90) ^a,b^ | <0.0001 |
| **Diabetes mellitus**, n (%) | 231 (28.73) ^b,c^ | 180 (22.19) ^a^ | 174 (21.64) ^a^ | <0.01 |
| **Depression**, n (%) | 249 (30.97) ^b,c^ | 145 (17.88) ^a,c^ | 104 (12.94) ^a,b^ | <0.0001 |

UGS, usual gait speed; KRW, Korean won; SGDS-K, Korean version of the Geriatric Depression Scale-Short Form; BMI, body mass index; SBP, systolic blood pressure; DBP, diastolic blood pressure; T-Chol, total cholesterol; HDL-C, high-density lipoprotein cholesterol; TG, triglyceride; FBG, fasting blood glucose; ^a^, *p*<0.05 vs. low UGS; ^b^, *p*<0.05 vs. mid UGS; ^c^, *p*<0.05 vs. high UGS.
