# Supplementary material for: Usual gait speed is inversely associated with depression in middle-aged and older adults: A cross-sectional study in Korea
Source: PLoS One. 2026 Feb 9;21(2):e0338458. doi: 10.1371/journal.pone.0338458 (PMC12885282; doi:10.1371/journal.pone.0338458)
Supplement: S2 Table — (DOCX) [file pone.0338458.s002.docx]

**S2 Table.** Odds ratios for depression prevalence according to sex-specific tertiles of the UGS in the various subgroups.

| **Subgroups** | **N** | **Depression**, n (%) | **Sex-specific UGS tertiles** | | | ***p* for interaction** |
| --- | --- | --- | --- | --- | --- | --- |
|  |  |  | **Depression**, n (%) | | **OR** (95% CI)  Low UGS vs. High UGS |  |
|  |  |  | **Low UGS** | **High UGS** |  |  |
| **Age** (years) |  |  |  |  |  |  |
| <75 | 1,193 | 213 (17.85) | 120 (27.84) | 93 (12.20) | 0.49 (0.35–0.69) ^****^ | 0.28 |
| ≥75 | 415 | 140 (33.73) | 129 (34.58) | 11 (26.19) | 0.80 (0.37–1.72) |  |
| **Sex** |  |  |  |  |  |  |
| Male | 709 | 96 (13.54) | 71 (20.06) | 25 (7.04) | 0.54 (0.31–0.96) ^*^ | 0.29 |
| Female | 899 | 257 (28.59) | 178 (39.56) | 79 (17.59) | 0.54 (0.36–0.82) ^**^ |  |
| **Marital status** |  |  |  |  |  |  |
| Divorced/widowed/single | 345 | 129 (37.39) | 103 (41.70) | 26 (26.53) | 0.69 (0.37–1.29) | 0.28 |
| Married/partnered | 1,263 | 224 (17.74) | 146 (26.21) | 78 (11.05) | 0.50 (0.34–0.74) ^***^ |  |
| **Educational level** |  |  |  |  |  |  |
| ≤Middle school | 1,176 | 307 (26.11) | 230 (33.77) | 77 (15.56) | 0.55 (0.38–0.79) ^**^ | 0.68 |
| ≥High school | 432 | 46 (10.65) | 19 (15.45) | 27 (8.74) | 0.61 (0.29–1.31) |  |
| **Household income** |  |  |  |  |  |  |
| <3 (million KRW/month) | 1,345 | 331 (24.61) | 244 (32.53) | 87 (14.62) | 0.50 (0.35–0.70) ^****^ | 0.16 |
| ≥3 | 263 | 22 (8.37) | 5 (9.26) | 17 (8.13) | 0.89 (0.19–4.17) |  |
| **Current drinking habit** |  |  |  |  |  |  |
| No | 1,033 | 255 (24.69) | 184 (34.20) | 71 (14.34) | 0.54 (0.36–0.81) ^**^ | 0.98 |
| Yes | 575 | 98 (17.04) | 65 (24.44) | 33 (10.68) | 0.56 (0.31–1.00) |  |
| **Smoking status** |  |  |  |  |  |  |
| Never | 1,434 | 319 (22.25) | 222 (30.88) | 97 (13.57) | 0.59 (0.42–0.84) ^**^ | 0.21 |
| Ever | 174 | 34 (19.54) | 27 (31.76) | 7 (7.87) | 0.31 (0.11–0.93) ^*^ |  |
| **Regular exercise** |  |  |  |  |  |  |
| <150 min/week | 1,346 | 319 (23.70) | 228 (31.93) | 91 (14.40) | 0.58 (0.41–0.83) ^**^ | 0.36 |
| ≥150 min/week | 262 | 34 (12.98) | 21 (23.33) | 13 (7.56) | 0.48 (0.19–1.22) |  |
| **BMI** (kg/m^2^) |  |  |  |  |  |  |
| <25 | 941 | 212 (22.53) | 152 (32.69) | 60 (12.61) | 0.51 (0.33–0.78) ^**^ | 0.24 |
| ≥25 | 667 | 141 (21.14) | 97 (28.61) | 44 (13.41) | 0.65 (0.39–1.09) |  |
| **Hypertension** |  |  |  |  |  |  |
| No | 752 | 140 (18.62) | 90 (28.39) | 50 (11.49) | 0.45 (0.27–0.76) ^**^ | 0.64 |
| Yes | 856 | 213 (24.88) | 159 (32.65) | 54 (14.63) | 0.62 (0.40–0.95) ^*^ |  |
| **Diabetes mellitus** |  |  |  |  |  |  |
| No | 1,203 | 258 (21.45) | 173 (30.19) | 85 (13.49) | 0.61 (0.42–0.90) ^*^ | 0.40 |
| Yes | 405 | 95 (23.46) | 76 (32.90) | 19 (10.92) | 0.42 (0.21–0.84) ^*^ |  |

UGS, usual gait speed; OR, odds ratio; CI, confidence interval; BMI, body mass index; ^*^, *p*<0.05; ^**^, *p*<0.01; ^***^, *p*<0.001; ^****^, *p*<0.0001. Adjusted for age, sex, drinking, smoking, educational level, marital status, household income, BMI, regular exercise, hypertension, and diabetes mellitus.
